# Quantifying the triboelectric series

**DOI:** 10.1038/s41467-019-09461-x

**Published:** 2019-03-29

**Authors:** Haiyang Zou, Ying Zhang, Litong Guo, Peihong Wang, Xu He, Guozhang Dai, Haiwu Zheng, Chaoyu Chen, Aurelia Chi Wang, Cheng Xu, Zhong Lin Wang

**Affiliations:** 1 School of Materials Science and Engineering, Georgia Institute of Technology, Atlanta, GA 30332-0245 USA; 2 Key Laboratory of Thermo−Fluid Science and Engineering, Ministry of Education, Xi’an Jiaotong University, 710049 Xi’an, Shaanxi Province People’s Republic of China; 3 School of Materials Science and Engineering, China University of Mining and Technology, 221116 Xuzhou, People’s Republic of China; 4 Beijing Institute of Nanoenergy and Nanosystems, Chinese Academy of Sciences, 100083 Beijing, People’s Republic of China

## Abstract

Triboelectrification is a well-known phenomenon that commonly occurs in nature and in our lives at any time and any place. Although each and every material exhibits triboelectrification, its quantification has not been standardized. A triboelectric series has been qualitatively ranked with regards to triboelectric polarization. Here, we introduce a universal standard method to quantify the triboelectric series for a wide range of polymers, establishing quantitative triboelectrification as a fundamental materials property. By measuring the tested materials with a liquid metal in an environment under well-defined conditions, the proposed method standardizes the experimental set up for uniformly quantifying the surface triboelectrification of general materials. The normalized triboelectric charge density is derived to reveal the intrinsic character of polymers for gaining or losing electrons. This quantitative triboelectric series may serve as a textbook standard for implementing the application of triboelectrification for energy harvesting and self-powered sensing.

## Abstract (web summary)

Triboelectric charging is a well-known phenomenon, but triboelectric polarization has only been ranked qualitatively. Here the authors develop a quantified triboelectric series for a wide range of polymers by measuring triboelectric charge density with respect to a liquid metal at well-defined conditions.

## Introduction

Driven by a diverse set of communities with unique and assorted requirements, the Materials Genome Initiative has attracted great attention globally to yield the new methods, metrologies, and capabilities necessary for accelerated materials development. The availability of high-quality materials data, including quantitative characterization and interoperability standards for material properties is crucial to achieving the advances of materials in multi-fields in science, engineering, and designs^1,2^. The triboelectric effect is a type of contact-induced electrification, owing to which a material would become electrically charged after it comes into frictional contact with another dissimilar material^3–7^. It is a natural effect for any material, and a general cause of everyday electrostatics, based on which electricity was discovered a few centuries ago. Materials are oppositely charged after physical contact, and the strength of the charges are different for different materials^8–10^. The only available tool that describes triboelectrification of materials is a triboelectric series, which is a list of materials behavior regarding their general trend of triboelectrification behavior without numerical data in the current existing form.

The triboelectric series ranks various materials according to their tendency to gain or lose electrons, which reflects the natural physical property of materials. Static electricity occurs when there is an excess of positive or negative charges on an object’s surface by rubbing certain materials together. The position of the material in the triboelectric series determines how effectively the charges will be exchanged. Normally, the build-up of static electricity would be undesirable because it can result in product failure or a serious safety hazard^11,12^ caused by electrostatic discharge and/or electrostatic attraction. This series can be used to select materials that will minimize static charging to prevent the electrostatic discharge or electrostatic attraction. Still, there are some practical applications^10,13^. Triboelectrification has found renewed interest recently as it has been used for fabricating triboelectric nanogenerators (TENGs), which have been used for efficiently converting mechanical agitations into electric signals for energy harvesters^14,15^, self-powered sensors^16,17^, and flexible electronics^14,18–20^. A large variety of materials have been chosen to fabricate the TENGs; the triboelectric series would help to show which pair of materials may work the best to create intentionally large static electricity by rubbing two materials in the same section in order to enhance the performance of TENGs.

Wilcke^21^ set-up the first triboelectric series in which about ten kinds of common materials were listed in the order of polarity. The series was further expanded by Shaw^22^ and Henniker^3^ by including natural and synthetic polymers, and showed the alteration in the sequence depending on surface and environmental conditions. Chun and colleagues^23^ made a tribo-charger set to measure the charge density of charged particles by using the charge-to-mass ratio of waste plastics, but this method is not acceptable for quantifying the surface charge density of a general flat material. Lee et al.^24^ tested different materials at low surface-to-surface force by a surface voltmeter to measure the charge affinity values, however, the charge transfer measurement has some limitations, for example the value unpredictably depends on the surface texture of the materials and the applied pressure^25^. Owing to complexities like humidity^26^, surface roughness^27^, temperature^28,29^, force or strain^30,31^, and other mechanical properties of materials involved in the experiment, different researchers received disjoined results in determining the rank of materials in the triboelectric series. By summarizing the existing literature, as triboelectrification is a two-materials interface-surface phenomenon and strongly depends on the contact of the two surfaces, it lacks a standard method that can accurately quantify the triboelectric charge density (TECD) of a general material with minimum uncertainty^32^. This study allows a figure-of-merit of TENGs to be quantified, and may impact other practical applications of the triboelectric effect.

Here, the triboelectric series of various polymers is quantitatively standardized by measuring the TECD with respect to a liquid metal, which is soft and shape adaptable to ensure an ideal surface contact with the material. The TECD is quantitatively measured in a glove box under well-controlled conditions, with fixed temperature, pressure and humidity. By contacting and separating with the liquid metal, the contact pressure could be kept the same, and the contact intimacy could be greatly enhanced so that it is able to achieve reliable values. The TECD is also normalized to show the intrinsic physical property of the materials.

## Results

### Principle for measuring the triboelectric charge density

**Fig. 1:**
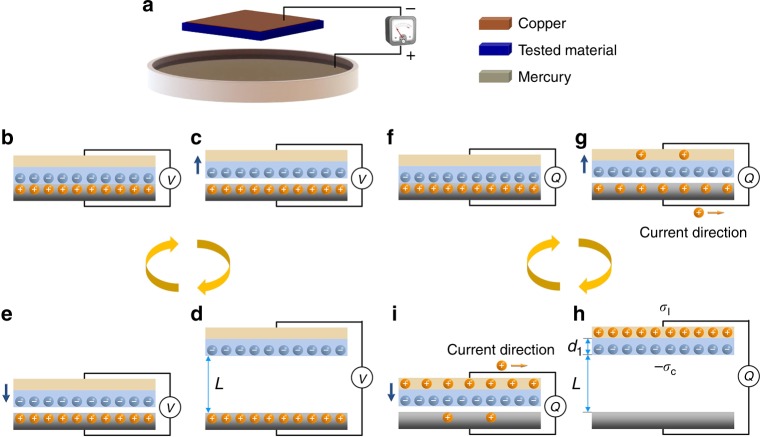
Principle for measuring the triboelectric charge density. **a** Simplified model of the measurement method. The tested material contacts the liquid metal of mercury, and then separates periodically. The positive electrode of the electric meter is connected to the mercury, and the negative is connected to the copper electrode. **b**–**e** The Theoretical model under open-circuit condition. **b** The contact electrification causes charge transfer between materials, the charges coincide at the same plane. The system has no net charge, there is no potential difference. **c** Voltage is generated between the two electrodes. Suppose the polymer has a strong capacity to absorb electrons, when the materials are separated, the polymer has negative charges, mercury has positive charges. Therefore, the potential difference is created between the two parts. **d** The potential reaches the maximum when the gap reaches certain distance *L*. The copper electrode is only influenced by the electric fields of the charges on the surface of the polymer. **e** When the polymer approaches the mercury, voltage drops due to the combined influence of the two electric fields. Finally, they are fully contacted, there is no voltage between the two electrodes (back to **b**). **f**–**i** The theoretical model under a short-circuit condition. **f** The two materials fully contact each other, there is no potential difference. **g** When the materials are separated, the negative charges on the surface of the polymer induce positive charges in copper, as copper and mercury are electronically connected, the positive charges in mercury flow into the copper side. **h** Approximately all charges flow into copper side to equalize the potential difference when the gap reaches certain distance *L*. **i** When the sample approaches the mercury, the negative charges on the surface of the polymer induces positive charges in mercury, the positive charges flow from copper to mercury until the charges are neutralized finally at the same plane when they are fully contacted (back to **f**)

The performance of triboelectric is influenced by the contact intimacy and the contact pressure. Solid materials have a low contact intimacy due to the nanometer-to-micrometer-level surface roughness^27^, therefore, the measurement between solid-solid contacts was not able to obtain a reliable TECD value. Another issue influencing the measurement result is the pressure applied between materials because of the unpredictable contact between two solid surfaces. Different materials have different mechanical properties (i.e., elasticity, hardness, stiffness), it is difficult to apply the same pressure between the solid materials all the time. To overcome this limitation of the contact between solid-solid interfaces and to build the measuring matrix for the triboelectric performance of materials, liquid metals of mercury (electronic grade 99.9998%) is utilized as the other triboelectrification material^33^. It is expected that, by using the liquid metal, the contact area is maximized as the liquid metal is shape-adaptive to solid surfaces. Moreover, mercury is a heavy metal and has large surface tension that the contact angle between mercury and tested materials are large (>130°) (Supplementary Fig. 1), unlike Galinstan^18^, the droplets tend to repel rather than adhere to the tested material surface during the contact-separation process, and the natural surface morphology differences would not have much impact on the measured results (Supplementary Fig. 2). Therefore, it leads to more reliable measurement results. As mercury dissolves most metals, a platinum wire (99.9% trace metals basis) was inserted into the mercury to connect the shield electric wire and mercury. The tested materials were placed in parallel facing to the surface of mercury (Fig. 1a).

The principle for measuring the TECD is based on the mechanism of TENG by coupling of contact electrification and electrostatic induction^34,35^. Figure 1 depicts the working processes under open-circuit (Fig. 1b–e) and short-circuit conditions (Fig. 1f–i). When two objects come into contact with each other, surface charge transfer takes place at the contact area due to contact electrification, resulting in one object gaining electrons on its surface, and another object losing electrons from its surface. As they are only confined to the surface, charges with opposite signs coincide at the same plane, there is no electric potential difference between the two electrodes (Fig. 1b). When the polymer and liquid metal are separated (Fig. 1c), there is no current flow under open-circuit condition, the copper metal electrode has no net charge, while the liquid metal electrode has positive charges caused by contact electrification. A potential difference is then established between the two electrodes as the opposite triboelectric charges are separated. When the gap distance raises up to a certain height of *L* after separating, which is chosen to be ~10 times the thickness of the materials, the open-circuit voltage reaches the maximum value (Fig. 1d). When the polymer is pushed down close to the liquid metal (Fig. 1e), the potential difference almost diminishes, and finally charges with opposite signs coincide at the same plane again (Fig. 1b).

If the two electrodes are electrically short-circuited (under coulombs measurement or amps measurement), the potential difference drives electrons to move from one electrode to the other to equalize their potential. When the polymer is lifted above the liquid surface, the negative surface charges on the polymer side would induce positive charges at the copper electrode side. As the copper and liquid metal are electrically connected, the positive charges on the liquid metal side would flow into the copper side (Fig. 1g). When the gap distance is above the height of *L*, the charges caused by contact electrification would be approximately fully transferred to the Cu electrode as the triboelectric charges (Fig. 1h) (see the theoretical estimation below). When the polymer is pushed downward toward the liquid metal, the negative charges on the surface of polymer would reduce the potential of the liquid metal side, resulting in the backflow of the positive charges from the Cu electrode (Fig. 1j). When the polymer and liquid metal are brought into contact, the two opposite charges are located at the same plane, there is no current flow after they fully contact (Fig. 1g). The TECD is measured by quantifying the total charges transferring between the Cu electrode and the liquid metal, as given by the following theoretical model.

For simplicity, a conductor-to-dielectric parallel-plate model is presented (Fig. 1). After their physical contact, the surfaces of the two have opposite static charges at a surface charge density *σ*_c_^36^. The surface charge density on the dielectric is fixed but the capacitance of the system changes during mechanical triggering^37^, so the density of induced static charge (*σ*_*I*_(*L*, *t*)) transferred between electrodes is the function of the gap distance *L*(*t*) when the value of *L* is low (*L* ≪ 10*d*_1_). From Gauss theorem, the electric field strength in the media and gap are approximately given by if the edge effect is ignored:

1 $$\begin{array}{*{20}{c}} {E_1 = \frac{{\sigma _I(L,t)}}{{\varepsilon _1}}} \end{array}$$

2 $$\begin{array}{*{20}{c}} {E_{{\mathrm{air}}} = \frac{{\sigma _I(L,t) - \sigma _c}}{{\varepsilon _0}}} \end{array}$$

where the dielectric permittivity of the material is *ε*_1_, and its thickness is *d*_1_. The voltage between the two electrodes is

3 $$\begin{array}{*{20}{c}} {V = \frac{{\sigma _I(L,t)}}{{\varepsilon _1}}d_1 + \frac{{\sigma _I(L,t) - \sigma _c}}{{\varepsilon _0}}L} \end{array}$$

Under short-circuit condition, the two electrodes are electrically connected, so *V* = 0, then,

4 $$\begin{array}{*{20}{c}} {\sigma _I(L,t) = \frac{{L\sigma _c}}{{\frac{{d_1\varepsilon _0}}{{\varepsilon _1}} + L}}} \end{array}$$

From Eq. (4), if the separation distance is much larger than the thickness of the materials (*L* ≫ *d*_1_*ε*_0_/*ε*_1_), ideally, the charge density of free electrons on surfaces of the electrode *σ*_*I*_(*L*,*t*) is very close to the surface charge density *σ*_c_. In our measurements, *d*_1_ = 0.5 mm, *L* = 75 mm, and when *ε*_1_/*ε*_0_ ~ 2, the measured charge density *σ*_*I*_ is 99.67% of the surface charge density *σ*_c_. Therefore, the measured value of the charge density represents the TECD on the dielectric surface.

### Configuration of the triboelectric series measurement

**Fig. 2:**
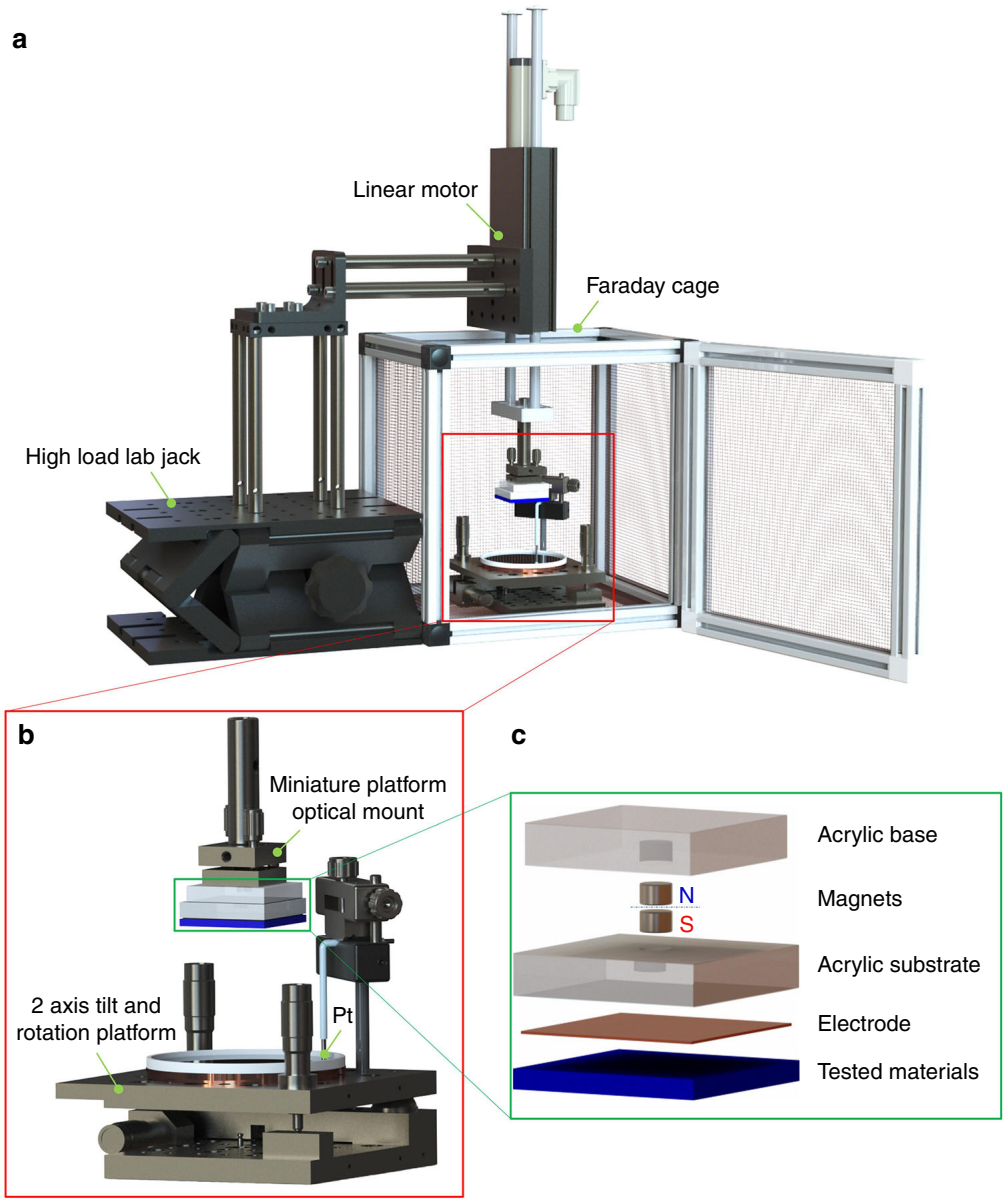
Experimental set-up for the triboelectric series measurement. The whole measurement was set in a glove box filled with ultra-high purity of nitrogen gas at fixed temperature, pressure, and humidity. The linear motor was settled on a high-load lab jack (**a**). The height of the sample to contact the liquid metal can be finely adjusted by both the high-load lab jack and a linear motor. The static part has the liquid metal as the electrode. The motion part consists of the tested sample, and it is controlled by the linear motor (**b**). An acrylic base is attached to the end of the linear motor. A magnet is engraved into the acrylic base to attract another magnet engraved in the sample substrate. Each sample consists of an acrylic substrate with the magnet, electrode, and the tested materials (**c**). Both the miniature platform optical mount and the two-axis tilt and rotation platform can adjust the orientations of the sample and liquid metal level

We have set-up a standardized measurement system for the TECDs of general materials, which is illustrated in Fig. 2, Supplementary Fig. 3 and Supplementary Movie 1. The set-up consists of the support part, a Faraday Cage, a static part, and a motion part. A height adjustable high-load lab jack supports a linear motor, and it could finely adjust the height position of the samples. To operate the system, the motion part is mounted on a linear motor and static part is mounted on a two-axis tilt and rotation platform, which is able to adjust the horizontal level of a Petri dish filled with mercury. The linear motor holds the samples, lift the samples up and down periodically with a displacement up to 75 mm to contact and separate with the liquid metal. The setting parameters of the linear motor are described in Supplementary Note 3, the route of the sample traveled is shown in Supplementary Fig. 4. A miniature platform optical mount is fixed at the end of the linear motor, which is designed to adjust the horizontal level of the sample. The details of the surface contact adjustments have been introduced in Supplementary Note 4. An acrylic sheet (1 in × 1 in) is fixed at the bottom of the optical mount, this insulator part separates the sample and linear motor to avoid any possible charge transfer to the tested sample.

The test materials are all commercial products purchased from vendors. The details of these materials and their sources are shown in the Supplementary Table 1. The tested materials were all cut into 38.1 × 38.1 mm, and then the surface was carefully cleaned with isopropyl alcohol (IPA) by cleanroom wipers and dried by a nitrogen gun. As some characteristics^38,39^ of materials could affect the measured TECD value, therefore, all materials remain their natural surface state as received without other further treatment. A mask with margins size of 2 mm was used to deposit 15 nm Ti and a thick layer of Cu (above 300 nm) on the samples as one electrode. The 2 mm edge was designed to avoid the short-circuit when the sample direct contacts with mercury. The coated side of the tested material was adhesive with the acrylic substrate by liquid epoxy glue. By using the liquid glue, the gas bubble could be removed carefully by pressing when the glue is in the liquid phase, and also fixed the acrylic substrate and tested materials when it is dried. Therefore, when the device was touching and separating with mercury, any noise signal generated between the acrylic substrate and copper could be avoided. Magnets were engraved into two acrylic sheets so that the two acrylic sheets would firmly be attached when they get in touch. These would make it simple and convenient to replace the samples, and the replaced sample also could return to the same position for measuring.

Noises may come from the AC from the glove box, light, and linear motor, and some electrified objects (Supplementary Fig. 5a). To limit the influence of noises, the samples were measured in a Faraday cage, which was connected to the ground outlet. Shield wires were used as connecting lines to screen the electric fields from the environment. The linear motor body’s parts were connected to ground together with any other objects that may be electrified to eliminate interference. Thus, the noise has been practically reduced to a low value (Supplementary Fig. 5b). The measurement system was set-up in a glove box so that the environmental conditions could be well-controlled. As the ionization of gas will greatly consume the electricity generated by contact electrification, the glove box was filled with ultra-high purity of nitrogen (99.999%). According to the Paschen’s law, the highest sparkover potential is obtained with nitrogen^40^, which could prevent arc-over between the two materials. The temperature was well-controlled in the glove box, which was measured to be 20 ± 1 °C. The pressure was fixed to be about 1 atm with additional about 1–1.5 in height of H_2_O, and the dew point ≤ −46 °C (0.43% in relative humidity (RH)). Anhydrous calcium chloride and phosphorus pentoxide powers were placed in two plates to absorb the water vapor in the glove box. Samples may have some water vapor on its surface, this would lead to measurement errors. Then, samples were placed in the glove box overnight before measuring to eliminate the vapor on the sample surface.

### The measured output signals and triboelectric charge densities

**Fig. 3:**
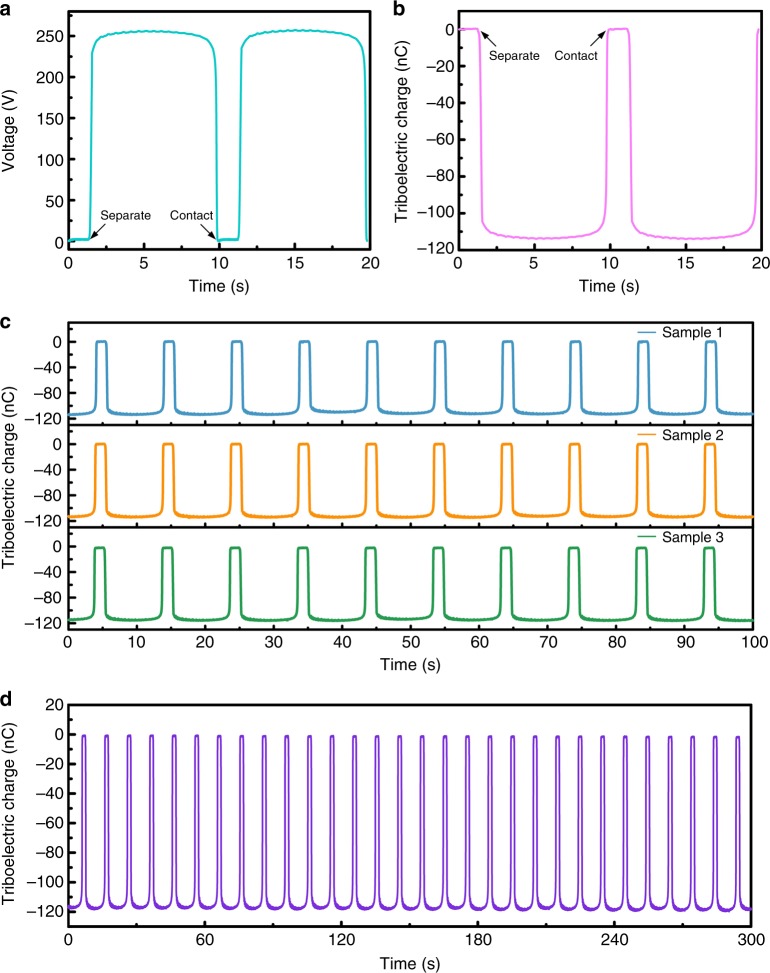
Typical measured signals. **a** A typical output of open-circuit voltage in two cycles of contact and separation. **b** Short-circuit transferred charge between the two electrodes in two cycles. **c** The measured charge transferred for three samples for the same material of PTFE. **d** Stability of the measured value over a relatively long time-period for many cycles

Typical signals of open-circuit voltage for polytetrafluoroethylene (PTFE) during the whole process are shown in Fig. 3a. When the PTFE was lifted up, the potential between the two electrodes reached up to 256.1 V. Typical signal of charges for PTFE for the whole process is shown in Fig. 3b. In this process, the Coulombs of electrons flow from the mercury electrode to the copper electrode is 113.84 nC. For the measurement, at least three samples were prepared for each type of materials to avoid measurement errors caused by device fabrication or operation errors during the test. Figure 3c shows the signals for three samples of PTFE. They are tested at different times, one in the morning, one in the evening and another in the next day. The results show that for the same material, the measured value is repeatable and reliable that it has no obvious change for the same material samples even measured at different times. The measured value has a good long-term stability, which is shown in Fig. 3d.

The transferred charges during the contact electrification were measured as the triboelectric charge, and the TECDs were calculated by the triboelectric charge over the area of the contact electrification surface. The varied charge densities measured by contacting tested materials with the liquid mercury indicates different capabilities of materials to receive electrons after contact electrification. Different samples may have their own initial surface charges, and the sample clean process before the measurement would also induce initial charges on the sample surface (Supplementary Note 6). To eliminate the initial surface charges of the samples, each sample had been kept running until it reaches its saturation level, the results were all recorded after the measured value reach its steady state (e.g., no obvious change after a hundred cycles).

**Fig. 4:**
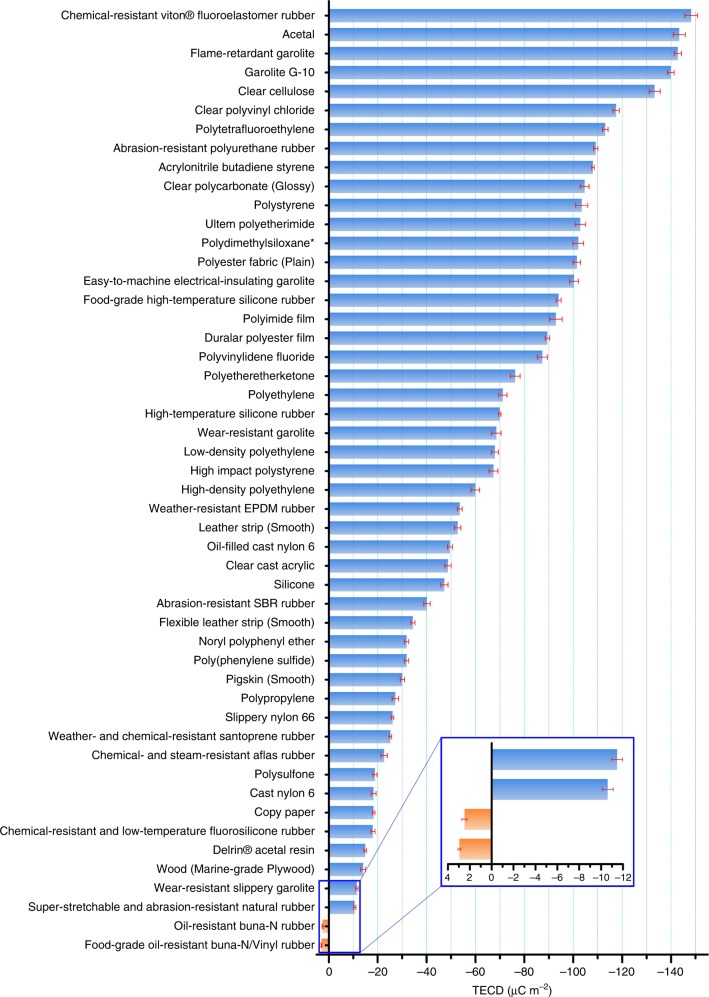
The quantified triboelectric series. The error bar indicates the range within a standard deviation. Source data are provided as a Source Data file

By summarizing the measurement results of over 50 materials, the triboelectric order is shown in Fig. 4. The data represent the average values of tests of at least three samples of the same materials. When the tested material is negatively charged after contact with mercury, the measured charge density is recorded as a negative value; when the material is positively charged after contact with mercury, it is recorded as a positive value. PTFE is most widely used in TENG devices, therefore, we can consider the TECD of PTFE with a thickness of 20 mil (~0.5 mm) as the reference TECD. Then the normalized triboelectric charge densities $\alpha = \frac{\sigma }{{|\sigma _{{\mathrm{PTFE}}|}}}$ of measured materials are listed in Table 1. The measurement was done in reference to liquid metal mercury. This is the standard for all of the measurements, so that the density can be quantified. For a material with *α* > 0, then the material is more positive than the reference liquid mercury; if *α* < 0, then this material is more negative than the reference liquid mercury. Materials that are farther apart on the list create a stronger charge when rubbed together. Materials near to each other on the series may exchange little charge (Supplementary Note 7). Dielectric-on-dielectric and metal-on-dielectric are mainly two types of pairs for TENG, so some common metals have been also tested by using the metal to contact and separate with various polymers to present the ranking of metals in triboelectric series as shown in Supplementary Table 2 for reference. It is worthy to note that triboelectricity is a two body problem that must have two materials that rub with each other. The sign of the triboelectric charges is relative to the counterpart. This means that the triboelectric series we proposed to use is only a qualitative estimation without mentioning what the counter material is.

**Table 1:** Triboelectric series of materials and their triboelectric charge density (TECD)

Materials	Abbr.	Average TECD (μC m^−2^)	STDEV	*α*
Chemical-Resistant Viton® Fluoroelastomer Rubber		−148.20	2.63	−1.31
Acetal		−143.33	2.48	−1.27
Flame-retardant garolite		−142.76	1.49	−1.26
Garolite G-10		−139.89	1.31	−1.24
Clear cellulose		−133.30	2.28	−1.18
Clear polyvinyl chloride	PVC	−117.53	1.31	−1.04
Polytetrafluoroethylene	PTFE	−113.06	1.14	−1.00
Abrasion-resistant polyurethane rubber		−109.22	0.86	−0.97
Acrylonitrile butadiene styrene	ABS	−108.07	0.50	−0.96
Clear polycarbonate (Glossy)	PC	−104.63	1.79	−0.93
Polystyrene	PS	−103.48	2.48	−0.92
Ultem polyetherimide	PEI	−102.91	2.16	−0.91
Polydimethylsiloxane*	PDMS	−102.05	2.16	−0.90
Polyester fabric (Plain)		−101.48	1.49	−0.90
Easy-to-machine electrical-insulating garolite		−100.33	1.79	−0.89
Food-grade high-temperature silicone rubber		−94.03	0.99	−0.83
Polyimide film	Kapton	−92.88	2.58	−0.82
DuraLar polyester film	PET	−89.44	0.86	−0.79
Polyvinylidene fluoride	PVDF	−87.35	2.06	−0.77
Polyetheretherketone	PEEK	−76.25	1.99	−0.67
Polyethylene	PE	−71.20	1.71	−0.63
High-temperature silicone rubber		−69.95	0.50	−0.62
Wear-resistant garolite		−68.51	1.99	−0.61
Low-density polyethylene	LDPE	−67.94	1.49	−0.60
High impact polystyrene		−67.37	1.79	−0.60
High-density polyethylene	HDPE	−59.91	1.79	−0.53
Weather-resistant EPDM rubber		−53.61	0.99	−0.47
Leather strip (Smooth)		−52.75	1.31	−0.47
Oil-filled cast nylon 6		−49.59	0.99	−0.44
Clear cast acrylic	PMMA	−48.73	1.31	−0.43
Silicone		−47.30	1.49	−0.42
Abrasion-resistant SBR rubber		−40.13	1.31	−0.35
Flexible leather strip (Smooth)		−34.40	0.86	−0.30
Noryl polyphenyl ether		−31.82	0.86	−0.28
Poly(phenylene Sulfide)	PPS	−31.82	0.86	−0.28
Pigskin (Smooth)		−30.10	0.86	−0.27
Polypropylene	PP	−27.23	1.31	−0.24
Slippery nylon 66		−26.09	0.50	−0.23
Weather- and chemical-resistant santoprene rubber		−25.23	0.50	−0.22
Chemical- and steam-resistant aflas rubber		−22.65	1.31	−0.20
Polysulfone		−18.92	0.86	−0.17
Cast nylon 6		−18.35	0.99	−0.16
Copy paper		−18.35	0.50	−0.16
Chemical-resistant and low-temperature fluorosilicone rubber		−18.06	0.86	−0.16
Delrin® Acetal Resin		−14.91	0.50	−0.13
Wood (marine-grade plywood)		−14.05	0.99	−0.12
Wear-resistant slippery garolite		−11.47	0.50	−0.10
Super-stretchable and abrasion-resistant natural rubber		−10.61	0.50	−0.09
Oil-resistant buna-N rubber		2.49	0.23	0.02
Food-grade oil-resistant buna-N/vinyl rubber		2.95	0.13	0.03

Note: STDEV refers to the standard deviation. The *α* refers to the measured triboelectric charge density of tested materials over the absolute value of the measured triboelectric charge density of the reference material. The material marked with an asterisk “*” means it has strong adhesion with mercury, a small drop of mercury is observed when it is separated with mercury. The measured TECD value may be a bit lower than its real value. Source data are provided as a Source Data file.

## Discussion

In summary, we have developed a universal method for standardized evaluation of the TECDs of various materials in a strictly controlled environment, providing a new “gene” of materials. The solid–liquid contact between tested materials and shape-adaptive liquid metal can lead to more accurate measurements. A quantitative triboelectric series was constructed based on the measured TECD. The normalized TECD reveals the materials’ capability to obtain or loss electrons after contact electrification. The quantified triboelectric series and the standards evaluation methods provided here develop the standards and techniques enabling acquisition, representation, and discovery of the very basic properties of materials, could set the foundation for the further applications and related fundamental research (i.e., the assessment of materials data, models, and simulations), which may support the Materials Genome Project for applications in energy harvesting, sensors, and human-machine interfacing.

## Methods

### Sample preparation

All tested materials were purchased from vendors. The materials were cut into 1.5 in × 1.5 in by a laser cutter (Universal Laser System, PLS6.75) or some other tools, and then were washed with cleanroom wipers, and dried by a nitrogen gun, and were kept in the glove box >12 h before measuring. Some materials, for example, copy paper, cannot be cleaned by any liquid, therefore they were only cleaned by flowing gas. The materials were then deposited with 10 nm Ti and a thick layer of copper (Cu) by E-beam evaporator (Denton Explorer). The tested materials were attached on the acrylic substrate by AB epoxy glue with the electrode facing the surface of the acrylic substrate. The gas bubble was carefully removed by pressing the samples on a cleanroom wiper hardly by hands, and kept pressing by a heavy metal block until the epoxy dried. A magnet was engraved on the other side of the acrylic substrate facing another magnet engraved in the acrylic base mounted on a linear motor (LinMot H01-23 × 86/160). The attraction between the magnets could firmly attach the sample with the linear motor. The sample could be lifted and pushed down automatically with the help of the linear motor. A plastic petri dish filled with the liquid mercury settled on a two-axis tilt and rotation platform (Newport M-37) was placed right below the sample. A shielded electric wire was soldered with a Platinum wire (Sigma Aldrich, 99.9% trace metals basis), and the Pt wire was inserted into the liquid mercury. Another shielded electric wire was connected with the Cu film. The two shielded electric wires were connected to two electrodes of the electrometer (Keithley 6514). The position of the sample was carefully adjusted by both of a high-load lab jack (Newport 281) and linear motor to make sure precisely right contact between the tested material and the liquid mercury; the angles of surfaces can be adjusted by both the two-axis tilt and rotation platform and the miniature platform optical mount (Newport P100-P), therefore, the surface would be able to be well contacted with the liquid metal.

All the experiments were measured in a glove box with an ultra-pure nitrogen environment (Airgas, 99.999%). The environmental condition was fixed at 20 ± 1 °C, 1 atm with additional about 1–1.5 in height of H_2_O and 0.43% RH.

## Supplementary information

Supplementary Information

Supplementary Movie 1

Source Data

## Data Availability

The datasets generated during and/or analyzed during the current study are available from the corresponding author. The source data underlying Table 1 are provided as a Source Data file.
