# Supplementary material for: Quantifying the triboelectric series
Source: Nat Commun. 2019 Mar 29;10:1427. doi: 10.1038/s41467-019-09461-x (PMC6441076; doi:10.1038/s41467-019-09461-x)
Supplement: Supplementary file 1 — Supplementary Information [file 41467_2019_9461_MOESM1_ESM.pdf]

Supplementary Information for

Quantifying the Triboelectric Series

Haiyang Zou[1]*, Ying Zhang[1,2]*, Litong Guo[1,3]*, Peihong Wang[1], Xu He[1], Guozhang Dai[1], Haiwu Zheng[1], Chaoyu Chen[1], Aurelia Chi Wang[1], Cheng Xu[1,3], Zhong Lin Wang[1, 4†]

[1] School of Materials Science and Engineering, Georgia Institute of Technology, Atlanta, Georgia 30332-0245, United States.

[2] Key Laboratory of Thermo–Fluid Science and Engineering, Ministry of Education, Xi'an Jiaotong University, Xi'an, Shaanxi Province 710049, P. R. China

[3] School of Materials Science and Engineering, China University of Mining and Technology, Xuzhou 221116, P.R. China

[4] Beijing Institute of Nanoenergy and Nanosystems, Chinese Academy of Sciences, Beijing 100083, P.R. China.

* These authors contributed equally to this work.

[†] Correspondence to: zhong.wang@mse.gatech.edu

This file includes:

Supplementary Note 1 to 7

Supplementary Figure 1 to 10

Supplementary Table 1 to 2

Supplementary Note 1 | Contact angle between mercury and tested materials

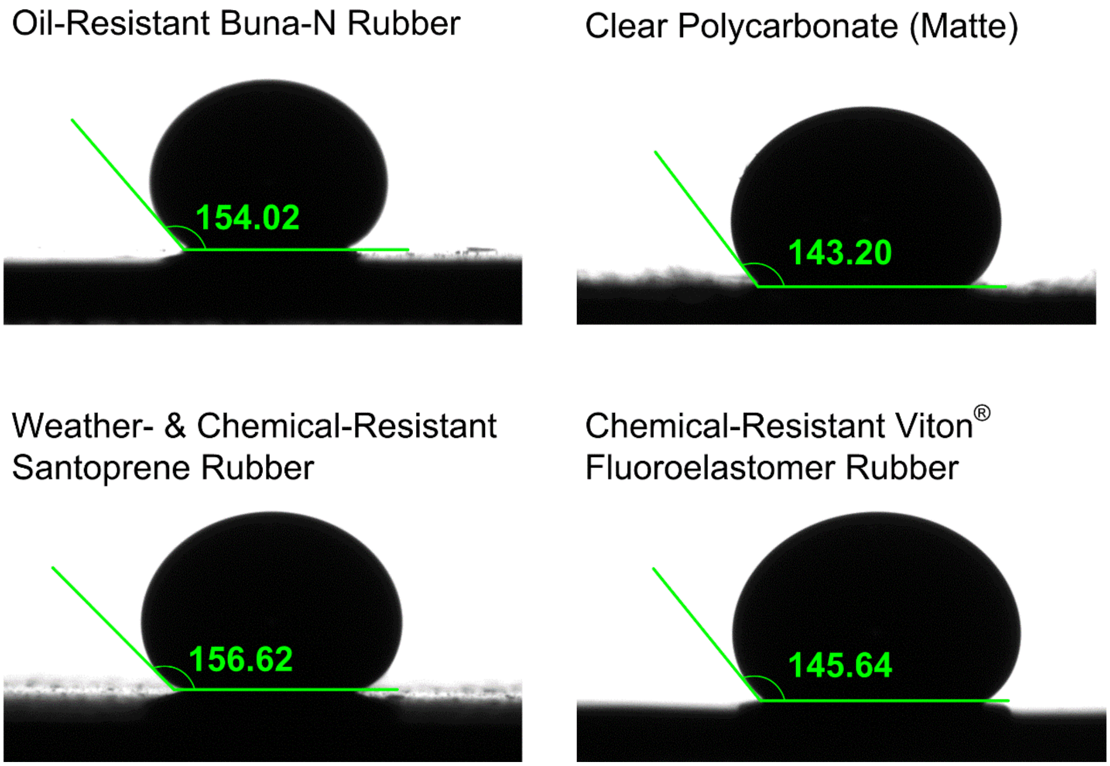

Supplementary Figure 1 | Contact angle between mercury and tested materials. It can be seen that the mercury has a large surface tension, the tested materials will be not wetted by the liquid metal. The mercury tends to repel when it touch the surfaces of tested materials. This is the reason that the mercury is the idea choice to be the other triboelectric material for measuring. The volume of the mercury measured on the surfaces is 25 mL.

Supplementary Note 2 | The effect of surface morphology

For the measurement on solid-solid interface, the surface morphology has a great influence on the measurement results. However, it is difficult to confirm each sample has perfect surface smoothness or the exactly the same surface roughness. Even the same patch of the same polymer made by the manufacturer, the surfaces of materials are different. Small scratches or other defects will make such measurement have measurement errors. Therefore, the liquid metal is used as one contact to greatly improve the contact intimacy. The measurement results of matte surface and smooth surface of clear polycarbonate is shown in Fig. S3. There is no obvious difference between the two measured values. The results demonstrate that the nanometer-to-micrometer-level surface roughness would not have a big influence on the measured results.

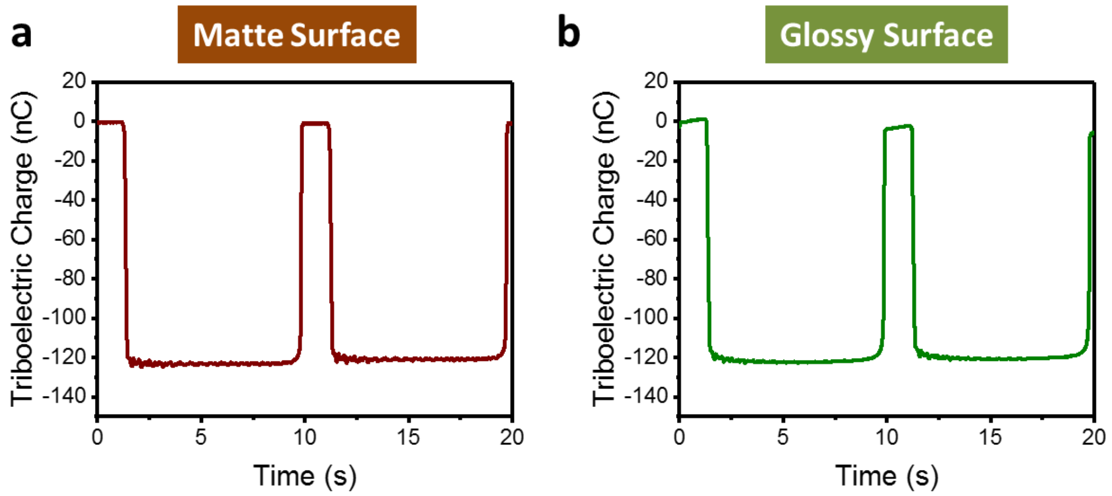

Supplementary Figure 2 | The comparison of Q-t curve of the material of clear polycarbonate with Matte surface (a) and glossy surface (b).

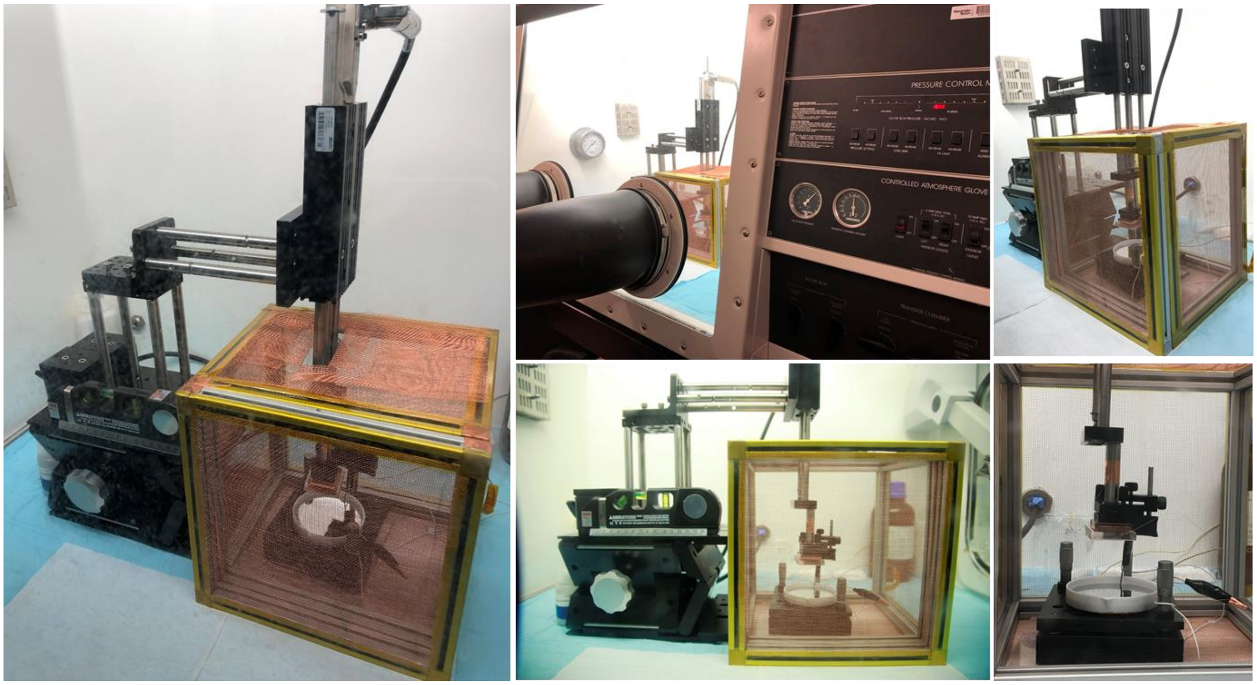

Supplementary Figure 3 | Photos of the measurement system.

Supplementary Note 3 | Linear motor parameters

The linear motor was set to rise at maximum speed of 0.02 m/s with acceleration of 0.1 m/s^2. The rise position wait time was 1 second. The fall maximum speed was set to 0.02 m/s with acceleration of 0.1 m/s^2. The position of maximum height to rise is 75 mm. The relationship of distance between the surface of the polymer and surface of the liquid metal is shown in Fig. S2.

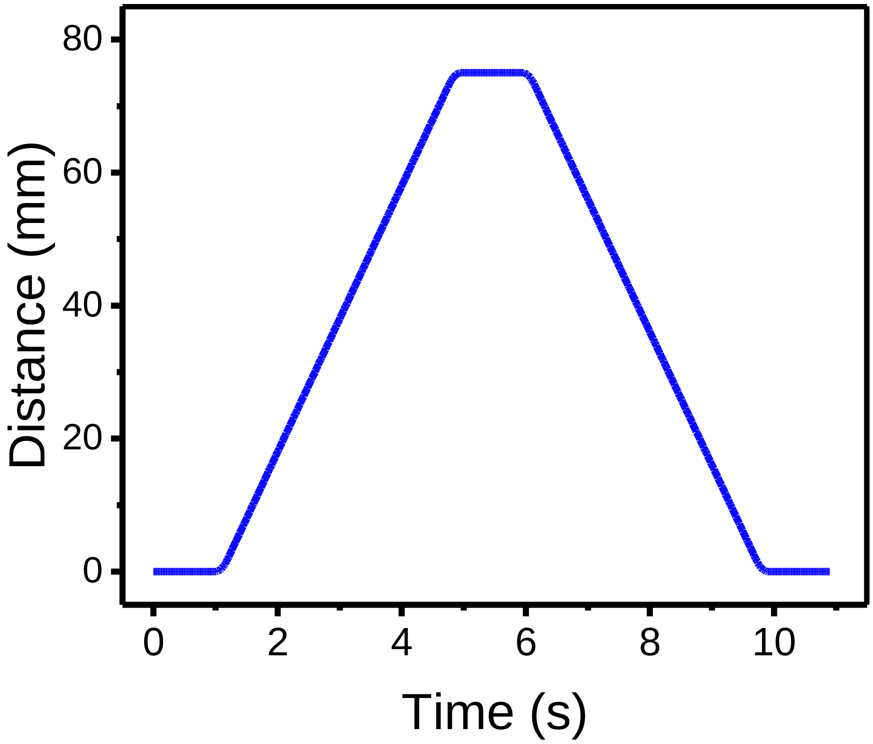

Supplementary Figure 4 | The motion track of the surface of the tested sample. The distance is the gap distance between the surface of the sample and the liquid level of the mercury.

Supplementary Note 4 | The surface contact adjustments

It is important to control the surface contact between two surfaces of tested sample and liquid metal to minimize measurement errors. If the surfaces are not well contacted, the measured value would not be reliable. There are two conditions that might happened, not fully contact or over contact. If the two surfaces are not horizontal leveled in the same plane, then only part of the tested sample was contacted with mercury, the reduced contact area will make the measured value smaller. To demonstrate this, we intentionally tilt the angle of the PTFE plane, so that only part (nearly 2/3 area) of the surface of PTFE contact the mercury. The signal is shown in Fig. S6, the measured value is much smaller than the value when the two planes are well contacted.

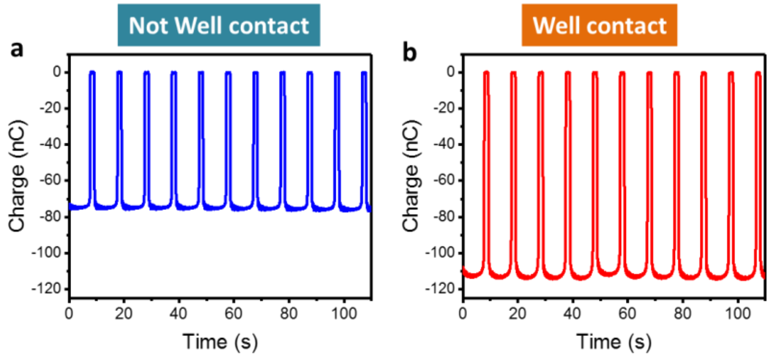

Supplementary Figure 6 | The comparison of Q-t curve of PTFE when the two planes are not fully contact (a) and well contact (b).

Secondly, if the tested sample was inserted deep into the mercury level, the edge of the acrylic substrate will contact mercury, will cause noise as shown in Fig. S7. We call it “cat ears”. When it is in over contact condition, the measured value is usually smaller than the value measured when they are well contacted.

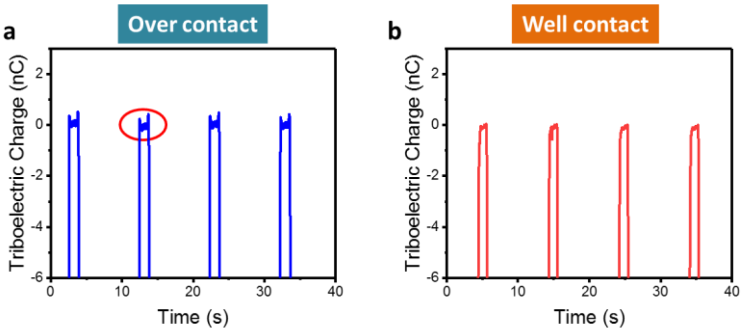

Supplementary Figure 7 | The comparison of Q-t curve of PTFE when the two planes are over contact (a) and well contact (b).

In our design, we have several measures to adjust the contact between the two objects including the height of samples to contact the liquid surface and the contact angle to allow the perfect contact with mercury. The height of samples can be controlled by a high load lab jack and linear motor. The resolution of the high load lab jack can be less than 1 mm, the linear motor can be 0.1 mm. Since the liquid phase of mercury has large surface tension, the two measures are enough to control the height of the sample to just contact the liquid level. The angle could be well controlled by a miniature platform optical mount and a 2 axis tilt & rotation platform. According to the information from the vendor, the sensitivity of the miniature platform is 8.3 arc sec, and the 2 axis tilt & rotation platform is 2 arc sec. These designs will help to ensure the two planes can be finely adjusted to have the good contact.

In our measurement, the basic logic to make the adjustments is very similar to the adjustment of the focusing for Scanning Electron Microscope (SEM). Firstly, coarse adjustments will be judges by eyes: through eyes, the height and angle of the sample will be adjusted to make sure the planes have contacted well. Secondly, fine adjustments will be made by try and error method. The reliable data always have the maximum value without “cat ears”. More importantly, we have fabricated more than 3 samples to check the results, so we were able to know the results were good or not. We would go back to measure the samples which is varied much from other samples of the same material, and fabricate new samples if it was needed.

Supplementary Note 5 | Noise control

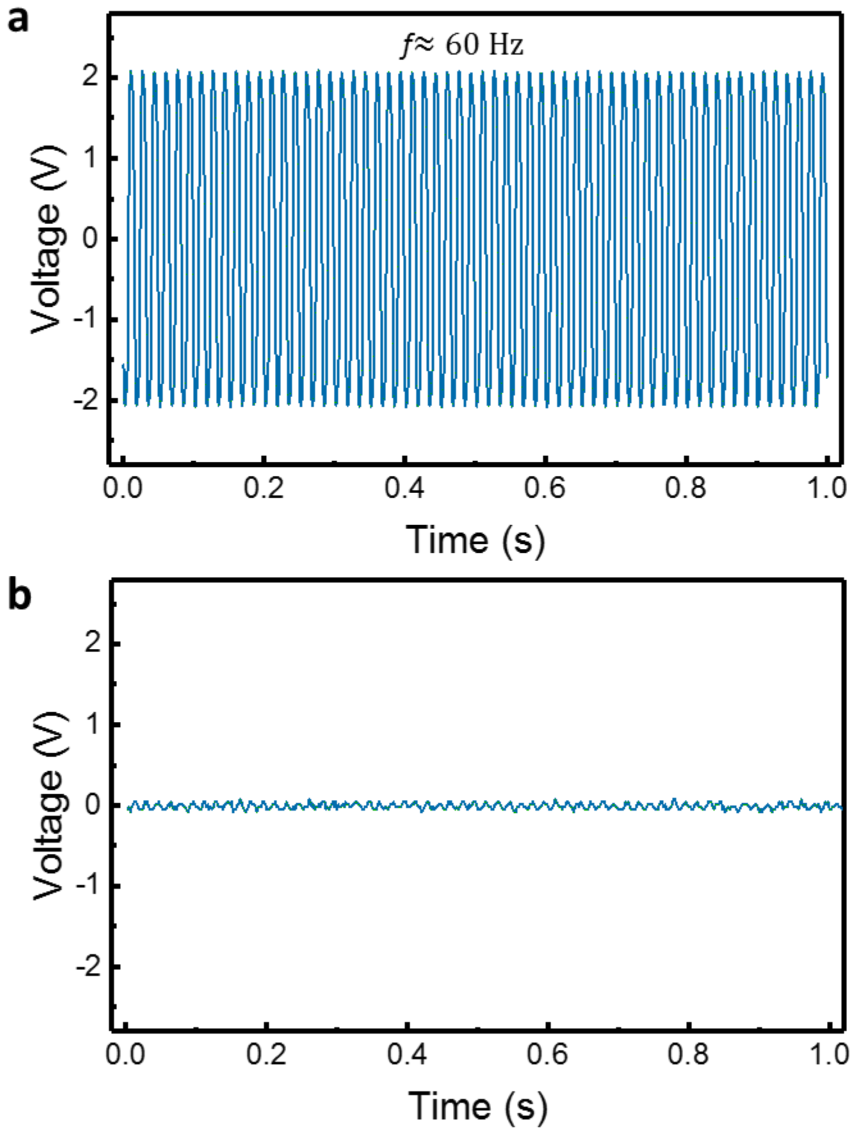

Supplementary Figure 5 | The signals of noise before (a) and after (b) the actions. (a) Without any steps to control the noise, the noise of the measuring system has a large value, which will greatly influence the measured results. The noise mostly like come from the AC electric power since the frequency is about 60 Hz. Many actions have been taken, which are listed as following: the charge is measured in a Faraday cage; shield wires are used as the connecting wires; the cage with all possible electrical charged objects including the body of the linear motor are connected with ground. The noise has been effectively reduced to a reasonable low value.

Supplementary Note 6 | Initial charge has to be avoided

The surfaces of the tested samples have initial surface charges at the beginning stage, especially when the surface has to be cleaned carefully before the test. Different samples would have different initial charges, even the sign of the charge would turn out to be opposite. For example, one of the samples - copy paper was measured to have positive charges at the beginning, then the positive charges gradually disappeared, and was measured to have negative charges finally. The copy paper was ultra-thin, it takes a longer time to eliminate the surface initial charge. The initial surface charge was found to cause the baseline shift. Finally, the copy materials will have a stable output value of their triboelectric charge after many cycles. This is similar to the result for slippery Nylon. The initial charges would be consumed by the impedance of the devices and measurement system.

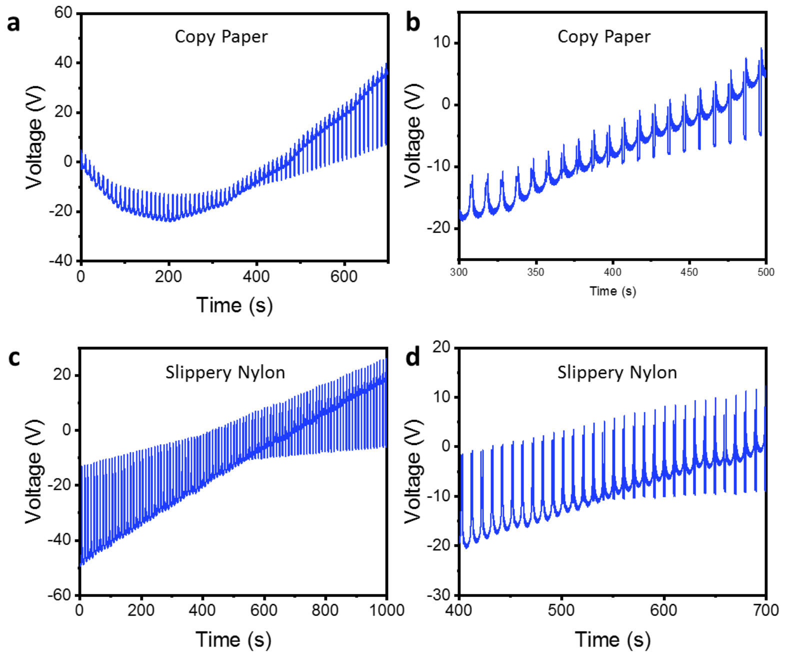

Supplementary Figure 8 | The influence of the opposite initial charge for the material of copy paper and slippery Nylon. (a, c) The copy paper and slippery Nylon originally had positive charges, but turned out to have negative charges after contacting with mercury many times. (b, d) The enlarge figures at the turning point when the charge was changed from positive to negative.

Supplementary Note 7 | The validity of the triboelectric series by cross-check

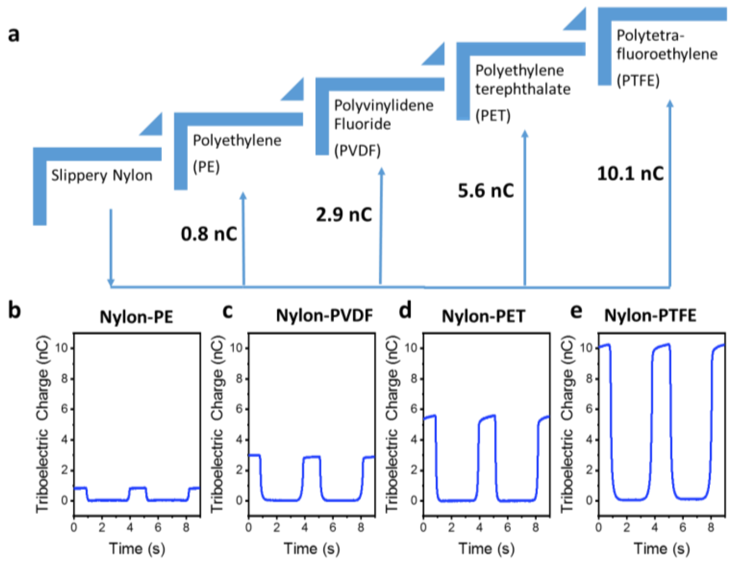

Supplementary Figure 9 | The charge transfer between Nylon and various materials. (a) The ranking for the tested materials in the triboelectric series. (b-e) The measured charge transfer between Nylon and PE (b), PVDF (c), PET (d), and PTFE (e).

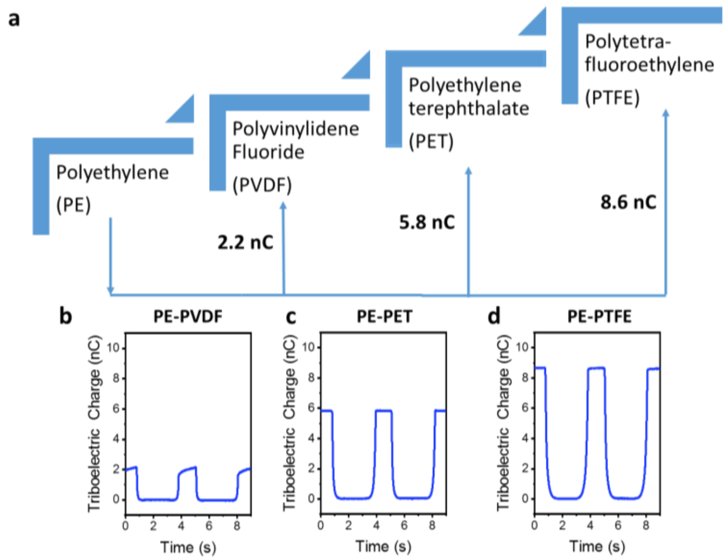

Supplementary Figure 10 | The charge transfer between Nylon and various materials. (a) The ranking for the tested materials in the triboelectric series. (b-e) The measured charge transfer between PE and PVDF (b), PET (c), and PTFE (d).

Supplementary Table 1 | The Information of Tested Materials

TE Series	Materials	Vendor	Catalog No.	Thickness (mil)
1	Chemical-Resistant Viton® Fluoroelastomer Rubber	Macmaster	86075K21	31.25
2	Acetal	Macmaster	5742T71	20
3	Flame-Retardant Garolite (Epoxy resins)	Macmaster	1331T29	20
4	Garolite G-10	Macmaster	9910T59	31.25
5	Clear Cellulose	Macmaster	8564K48	20
6	Clear Polyvinyl Chloride	Macmaster	8785K17	20
7	Polytetrafluoroethylene	Grainger	30FZ59	10
8	Abrasion-Resistant Polyurethane Rubber	Macmaster	2178T21	20
9	Acrylonitrile Butadiene Styrene	Macmaster	5751T71	20
10	Clear Polycarbonate (Glossy)	Macmaster	85585K45	20
11	Polystyrene	Macmaster	8734K31	31.25
12	Ultem PEI	Macmaster	7576K14	20
13	Polydimethylsiloxane	Fisher Scientific	NC9644388	20
14	Polyester Fabric (Plain)	Macmaster	8843K31	22
15	Easy-to-Machine Electrical-Insulating Garolite	Macmaster	8525K411	31.25
16	Food-Grade High-Temperature Silicone Rubber	Macmaster	86045K76	31.25
17	Polyidide Film	Mcmaster	7161T21	20
18	DuraLar Polyester Film	Sam Flax	306469	20
19	Polyvinylidene Fluoride	Macmaster	8675K25	20
20	Polyetheretherketone	Macmaster	8504K71	62.5
21	Polyethylene	Grainger	53TC08	20
22	High-Temperature Silicone Rubber	Macmaster	86435K15	20
23	Wear-Resistant Garolite	Macmaster	8491K1	31.25
24	Low-Density Polyethylene	Grainger	1YZV4	63
25	High Impact Polystyrene	Macmaster	8748K21	31.25
26	High-Density Polyethylene	Grainger	1ZAV5	64
27	Weather-Resistant EPDM Rubber	Macmaster	8610K42	62.5
28	Leather Strip (Smooth)	Macmaster	8706K52	78.125
29	Oil-Filled Cast Nylon 6	Macmaster	84845K111	250
30	Clear Cast Acrylic	Macmaster	8560K171	62.5
31	Silicone	Ecoflex	00-30	60
32	Abrasion-Resistant SBR Rubber	Macmaster	8634K41	62.5
33	Flexibel Leather Strip (Smooth)	Macmaster	8706K72	78.125
34	Noryl Polyphenyl Ether	Macmaster	8561K419	62.5
35	Poly(phenylene Sulfide)	Macmaster	1906T21	250
36	Pigskin (Smooth)	Macmaster	8724T11	27
37	Polypropylene	Grainger	22JL61	63
38	Slippery Nylon 66	Macmaster	8555K31	31.25
39	Weather- and Chemical-Resistant Santoprene Rubber	Macmaster	86215K11	31.25
40	Chemical- and Steam-Resistant Aflas Rubber	Macmaster	5499T11	31.25
41	Polysulfone	Macmaster	86735K71	375
42	Cast Nylon 6	Macmaster	85055K118	250
43	Copy Paper	Staples	135855	0.394
44	Chemical-Resistant and Low-Temperature Fluorosilicone Rubber	Macmaster	2183T2	31.25
45	Delrin® Acetal Resin	Macmaster	8573K281	31.25
46	Wood (Marine-grade Plywood)	Macmaster	1125T21	375
47	Wear-Resistant Slippery Garolite	Macmaster	2547K11	62.5
48	Super-Stretchable and Abrasion-Resistant Natural Rubber	Macmaster	85995K27	20
49	Oil-Resistant Buna-N Rubber	Macmaster	8635K541	31.25
50	Food-Grade Oil-Resistant Buna-N/Vinyl Rubber	Macmaster	8615K82	62.5

Supplementary Table 2 | The metals in Triboelectric series in polymers

Materials
Chemical-Resistant Viton® Fluoroelastomer Rubber
Acetal
Flame-Retardant Garolite
Garolite G-10
Clear Cellulose
Clear Polyvinyl Chloride
Polytetrafluoroethylene
Abrasion-Resistant Polyurethane Rubber
Acrylonitrile Butadiene Styrene
Clear Polycarbonate (Glossy)
Polystyrene
Ultem Polyetherimide
Polydimethylsiloxane*
Polyester Fabric (Plain)
Easy-to-Machine Electrical-Insulating Garolite
Food-Grade High-Temperature Silicone Rubber
Polyimide Film
DuraLar Polyester Film
Polyvinylidene Fluoride
Polyetheretherketone
Polyethylene
High-Temperature Silicone Rubber
Wear-Resistant Garolite
Low-Density Polyethylene
High Impact Polystyrene
High-Density Polyethylene
Au
Weather-Resistant EPDM Rubber
Leather Strip (Smooth)
Oil-Filled Cast Nylon 6
Clear Cast Acrylic
Silicone
Cu
Abrasion-Resistant SBR Rubber
Flexible Leather Strip (Smooth)
Ag
Poly(phenylene Sulfide)
Noryl Polyphenyl Ether
Pigskin (Smooth)
Polypropylene
Slippery Nylon 66
Weather- and Chemical-Resistant Santoprene Rubber
Chemical- and Steam-Resistant Aflas Rubber
Polysulfone
Copy Paper
Cast Nylon 6
Chemical-Resistant and Low-Temperature Fluorosilicone Rubber
Delrin® Acetal Resin
Wood (Marine-grade Plywood)
Wear-Resistant Slippery Garolite
Super-Stretchable and Abrasion-Resistant Natural Rubber
Hg
Oil-Resistant Buna-N Rubber
Food-Grade Oil-Resistant Buna-N/Vinyl Rubber

Note: Metals can induce triboelectric charges, but cannot kept charges at their surface like polymers. The charges are distributed in the whole body rather than the surface. The TECD of metals cannot be obtained by the proposed method. Firstly, mercury can dissolve most metals; secondly, the two metals of mercury and tested metals has no charge transfer. Since the metal-on-dielectric model in TENGs is important, here we adopted the traditional method of ranking the metals according to their tendency to gain or lose electrons. Here, the ranking of metals in triboelectric series is measured by contacting and separating various polymers and the pure tested metals (99.99%) deposited on substrates.
